# BENEFITS OF VOLUNTEERING ON RESILIENCE WITH AGING: A CASE STUDY

**DOI:** 10.1093/geroni/igac059.2165

**Published:** 2022-12-20

**Authors:** Sanghee Lee, Jinmoo Heo, Sanghee Chun

**Affiliations:** Korea University, Seoul, Seoul-t'ukpyolsi, Republic of Korea; Yonsei University, Seoul, Seoul-t'ukpyolsi, Republic of Korea; Brock University, St. Catharines, Ontario, Canada

## Abstract

As the study of volunteering among older adults continues to evolve, questions of the benefits of volunteering are of growing interest to many researchers. Volunteering may develop resilience in older adults as it can serve as a coping strategy as they recover from adverse events or other life challenges. We explored the perceived benefits of volunteering on resilience in later life among older adults who perform Korean traditional dance on voluntary basis. We used a qualitative design with a case study method. In this study, older adults’ volunteer dance performing was taken as a case. A case design enables researchers to understand social and cultural phenomenon in depth using a wide range of data collection and analysis methods. Thirteen volunteer performers of Korean traditional dance whose ages ranged between 61-74 years were recruited for in-depth interviews (11 females and 2 males). The analysis of the transcripts generated five themes related to the benefits of volunteering: (1) finding a sense of self-worth through serving others, (2) finding a sense of purpose, (3) experiencing gratitude, (4) renewing a younger self, and (5) building companionship. The findings of this study provided an empirical support showing how volunteering experience benefited resilience among the volunteers in the face of challenges associated with aging by maximizing positive physical, social, and psychological outcomes through involvement in voluntary dance performance. The findings also provide guidance for researchers and practitioners in positions to better serve older adults and thereby suggest volunteering as a resilience strategy in later life.

